# High‐Resolution Large‐Area Image Analysis Deciphers the Distribution of *Salmonella* Cells and ECM Components in Biofilms Formed on Charged PEDOT:PSS Surfaces

**DOI:** 10.1002/advs.202307322

**Published:** 2024-01-15

**Authors:** Sanhita Ray, Susanne Löffler, Agneta Richter‐Dahlfors

**Affiliations:** ^1^ AIMES – Center for the Advancement of Integrated Medical and Engineering Sciences at Karolinska Institutet and KTH Royal Institute of Technology Stockholm SE‐171 77 Sweden; ^2^ Department of Neuroscience Karolinska Institutet Stockholm SE‐171 77 Sweden

**Keywords:** biofilm, conductive polymers, ECM, image processing, PEDOT:PSS, redox, Salmonella

## Abstract

Biofilms, comprised of cells embedded in extracellular matrix (ECM), enable bacterial surface colonization and contribute to pathogenesis and biofouling. Yet, antibacterial surfaces are mainly evaluated for their effect on bacterial cells rather than the ECM. Here, a method is presented to separately quantify amounts and distribution of cells and ECM in *Salmonella* biofilms grown on electroactive poly(3,4‐ethylenedioxythiophene):polystyrenesulfonate (PEDOT:PSS). Within a custom‐designed biofilm reactor, biofilm forms on PEDOT:PSS surfaces electrically addressed with a bias potential and simultaneous recording of the resulting current. The amount and distribution of cells and ECM in biofilms are analyzed using a fluorescence‐based spectroscopic mapping technique and fluorescence confocal microscopy combined with advanced image processing. The study shows that surface charge leads to upregulated ECM production, leaving the cell counts largely unaffected. An altered texture is also observed, with biofilms forming small foci or more continuous structures. Supported by mutants lacking ECM production, ECM is identified as an important target when developing antibacterial strategies. Also, a central role for biofilm distribution is highlighted that likely influences antimicrobial susceptibility in biofilms. This work provides yet a link between conductive polymer materials and bacterial metabolism and reveals for the first time a specific effect of electrochemical addressing on bacterial ECM formation.

## Graphical abstract

This study shows how electroactive materials dynamically affect the structure and behavior of biofilms growing on their surface. It is shown, for the first time, that electrical charging of conducting polymer surfaces leads to changes in bacterial production of biofilm extracellular matrix while the amounts of bacterial cells growing on these surfaces remain unaffected.

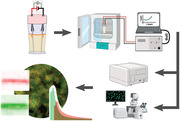

## 1 Introduction

Biofilms can be described as the houses and cities of the bacterial world.^[^ ^1^ ^]^ It is estimated that 40–80% of the bacterial species organize themselves in assemblies in which free‐living, so‐called sessile, bacteria are surrounded by a self‐produced extracellular matrix ECM), which is composed of functional protein aggregates, exopolysaccharides, and extracellular DNA.^[^ ^2^, ^3^ ^]^ The ECM creates a distinct microenvironment that protects the bacteria from environmental stressors, facilitates interactions between members of the community, and aid in resource capture.^[^ ^4^, ^5^ ^]^ Owing to the biofilm lifestyle, bacteria are important for the functioning of ecosystem, driving biogeochemical processes, nutrient cycling, and bioremediation.^[^ ^6^ ^]^ Conversely, biofilms related to bacterial infections are often extremely difficult to treat,^[^ ^7^, ^8^, ^9^ ^]^ and many industrial applications struggle with biofouling due to biofilm formation.^[^ ^10^, ^11^ ^]^

The ability to modulate biofilm formation is key to support its formation in a range of applications when beneficial, e.g. in waste‐water treatment, microbial fuel cells, and within the oral, intestinal, or cutaneous microbiome, or to hinder and eliminate biofilm formation when problematic, e.g. medical implants and prostheses. For long, researchers have tried to support or hinder biofilm formation by introducing surface modifications targeting the mechanisms of bacterial attachment.^[^ ^12^ ^]^ Since bacteria are very adaptive, they may overcome challenges posed by static surface properties. An alternative approach was taken as we developed systems influencing the availability of electron acceptors, aimed to target the metabolism of bacteria. In a pioneering study, we showed how *Salmonella* biofilm formation on surfaces coated with the redox‐active conjugated polymer poly(3,4‐ethylenedioxythiophene) (PEDOT) was modulated dependent on the electrochemical state.^[^ ^13^ ^]^ In this study, we electrodeposited PEDOT with the dopants chloride, dodecyl benzene sulfonate, and heparin, and showed that the effect of the dopant on biofilm formation was minimal. In a follow‐up study, we used a screen‐printed device using commercially available dispersion of PEDOT doped with polystyrene sulfonate (PSS), showing that bacteria secrete small, redox‐active compounds which cause reduction of the material.^[^ ^16^ ^]^ Taken together, results from these studies founded our hypothesis that bacteria use the electroactive material as electron sink, leading to bacterial growth and increased deposition of biomass on the oxidized material.

To decipher the effects of electroactive surface modulation on bacterial growth and production of ECM, novel tools are needed for visualization, identification, and quantification of the biomacromolecules of the ECM. Standard methods often rely on non‐specific fluorochromes, such as Calcofluor White, which due to its strong interaction with glycosidic bonds stains a variety of polysaccharides.^[^ ^17^ ^]^ Another common stain is Congo Red (CR) by which colony morphology of species and strains producing the functional amyloid protein curli are assessed.^[^ ^18^ ^]^ A variety of other amyloid and carbohydrate‐binding molecules have also been used.^[^ ^19^, ^20^ ^]^ The disadvantage common to these methods is that their use is restricted to endpoint assays, which hinder real‐time studies of biofilm formation. Recently, we developed a new group of easy‐to‐use, fluorescent tracer molecules, so‐called optotracers, which visualize and identify biomacromolecules in live biofilm assays.^[^ ^21^ ^]^ The commercialized version of this technique, available under the brand name EbbaBiolight, has been used to study *Salmonella* and *Escherichia coli* (*E. coli*) biofilm formation.^[^ ^22^, ^23^ ^]^ The technique was further developed into the first antibiofilm‐specific antibiotic susceptibility test (AST), visualizing and quantifying the effects of clinically relevant antibiotics on the growth of bacterial cells and the production of ECM components within biofilms.^[^ ^24^ ^]^ EbbaBiolight was also used to study nanoparticle diffusion in *Salmonella* ECM, and biofilm formation in the fungus *Candida albicans*.^[^ ^25^, ^26^ ^]^ In the latter case, EbbaBiolight stained intracellular amyloids with high quantum yield, whereas glucans in the yeast cell wall were labeled with lower quantum yield and appeared less bright in confocal fluorescence images. In other gram‐negative bacteria, such as *Burkholderia cenocepacia*, *Klebsiella pneumoniae* and *Pseudomonas aeruginosa*, EbbaBiolight has been used as a general ECM label without further specification of binding targets.^[^ ^27^, ^28^, ^29^, ^30^, ^31^ ^]^

In the biofilm lifestyle, bacteria can colonize abiotic surfaces, such as stainless steel,^[^ ^32^ ^]^ glass,^[^ ^33^, ^34^ ^]^ and polymers.^[^ ^13^, ^35^, ^36^ ^]^ The overall biomass of biofilms consists of bacterial cells together with a large fraction that is attributed to the extracellular polymeric matrix.^[^ ^37^ ^]^ Although the ECM composition varies between species, adhesins, amyloid‐forming proteins, and extracellular polysaccharides are ubiquitous components of most ECM.^[^ ^38^ ^]^ The amyloid curli and bacterially produced cellulose have been identified as important ECM components for *E. coli* and *Salmonella enterica* serovars Enteritidis (*S*. Enteritidis) and Typhimurium (*S*. Typhimurium).^[^ ^39^, ^40^ ^]^ The use of bacterial mutants unable to express curli (Δ*csgA*), cellulose (Δ*bcsA*), or both (Δ*csgD*) have aided in the characterization of the genetic regulatory networks controlling curli and cellulose expression.^[^ ^41^ ^]^ The availability of wild‐type (wt) and isogenic strains harboring mutations in genes encoding essential components of the ECM has made this collection of *S*. Enteritidis and *S*. Typhimurium strains very useful for research on biofilm formation.^[^ ^13^, ^21^, ^22^, ^24^, ^36^ ^]^

Here, we use EbbaBiolight 680 to analyze the individual contribution of bacterial cells and ECM in *Salmonella* biofilms formed on electroactive surfaces. We develop a biofilm reactor in which *Salmonella* form surface interface biofilms at the air‐liquid interface on electronically addressed PEDOT:PSS coated slides. By recording the electrical current, we monitor how bacterial biofilm formation affects electrochemical processes of unbiased, reduced, and oxidized surfaces. Conversely, we monitor whether the surfaces’ electrochemical state influences biofilm formation. Using fluorescence signals from a green fluorescence protein (GFP) specifying bacterial cells^[^ ^42^ ^]^ and ECM (EbbaBiolight 680), we apply spectrophotometric mapping to quantify the individual amounts of cells and ECM of surface interface biofilms. To map the distribution of cells and ECM, we develop a process for high‐resolution large‐area imaging analysis. These data, supported by studies of mutants unable to produce selected ECM biomacromolecules, help us to decipher the differential effect of electroactive surface modulation on bacterial cells and ECM on a gene regulatory level.

## 2 Results

### 2.1 Coating of PEDOT:PSS on ITO Produces Highly Wettable Films on Large Surface Area

**Figure 1:**
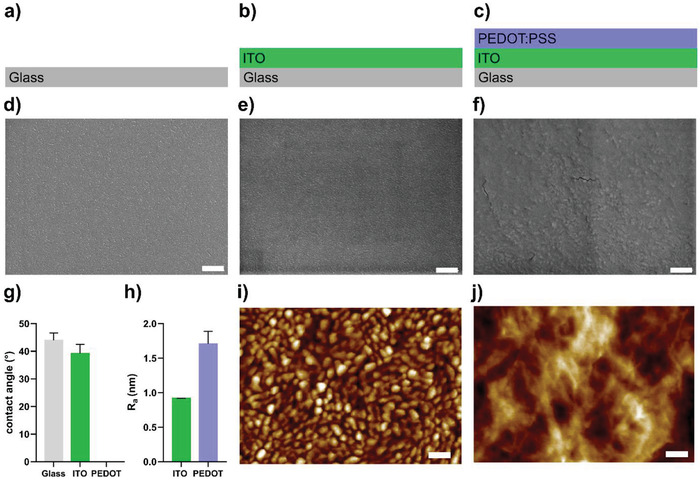
Characterization of PEDOT:PSS/ITO slides with full coverage and high wettability. a–c) Schematic representation of surfaces used to generate PEDOT:PSS/ITO slides: a) microscope slide made of glass, b) ITO slide, c) PEDOT:PSS/ITO slides. d–f) SEM showing the surface morphology of d) glass slide, e) ITO slide, and f) PEDOT:PSS/ITO slide (scale bar = 100 nm). g) Wettability measurement showing the contact angle of a water droplet on glass, the ITO slide (ITO) or PEDOT:PSS/ITO slides (PEDOT). h) Average roughness of ITO slides and PEDOT:PSS/ITO slides (PEDOT) obtained from AFM scans. i,j) AFM scans showing the morphology of i) ITO slides with scale bar = 50 nm and a height range of ±4.0 nm, and j) PEDOT:PSS/ITO slides with scale bar = 50 nm and a height range of ±6.5 nm. *n* = 1 for SEM, *n* = 2 for AFM.

To obtain an electroactive coating covering the whole surface of a standard microscope slide, we spin‐coated a dispersion of PEDOT:PSS onto plasma‐treated indium tin oxide (ITO) coated glass slides (ITO slides). To enable long‐term incubation of PEDOT:PSS coated ITO slides (PEDOT:PSS/ITO slides) in the aqueous environment of bacterial growth medium, we stabilized the PEDOT:PSS films by adding (3‐glycidyloxypropyl) trimethoxysilane (GOPS). GOPS cross‐links PEDOT:PSS via the sulfonic acid group of the excess PSS, maintaining oxidation level of PEDOT.^[^ ^43^ ^]^ To activate the crosslinker, the spin‐coated PEDOT:PSS/ITO slides were baked at 150 °C overnight. When analyzing the surface morphology by scanning electron microscopy (SEM), we used glass slides (**Figure** **1**a) and ITO slides (Figure 1b) for comparisons to the PEDOT:PSS/ITO slides (Figure 1c). When we applied scanning electron microscopy (SEM) to surfaces sputter‐coated with platinum (Pt), glass slides showed a typical clean morphology (Figure 1d), while ITO slides showed a uniform morphology with some granularity in the range of 10–20 nm (Figure 1e). The PEDOT:PSS/ITO slide contained a thick continuous layer of PEDOT:PSS with good coverage (Figure 1f). Some depressions and features in the range of 100–200 nm, as well as a few cracks from the baking process in the range of 10–20 nm, were visible. We performed wettability tests on glass, ITO and PEDOT:PSS/ITO slides. We found the contact angle of ITO slides to be in the same range as glass, whereas the PEDOT:PSS/ITO slide was highly wettable, with water droplets dispersing into the material within a few seconds (Figure 1g). Roughness analysis was performed using atomic force microscopy (AFM), with average roughness R_a_ summarized in Figure 1h. AFM imaging of the ITO slide showed the granularity typical for ITO (Figure 1i) and R_a_ of 0920±0001. For the PEDOT:PSS/ITO slide, the morphology aligned well with the model having two distinct yet highly interconnected phases consisting of PEDOT‐rich and PSS‐rich domains, as proposed by Volkov^[^ ^44^ ^]^ (Figure 1j). The R_a_ of PEDOT:PSS/ITO was 1.710±0.179 nm. The thickness of the PEDOT:PSS coating on the ITO slides was determined by spin‐coating the material as usual, but masking an area using Kapton tape, which was removed prior to analysis by atomic force microscopy (AFM). Scanning from the uncoated (masked) area to the coated area revealed a step height of 219 nm (Figure S1, Supporting Information).

### 2.2 Large Area PEDOT:PSS/ITO Surfaces Allow for Charge Storage When Electrically Addressed

**Figure 2:**
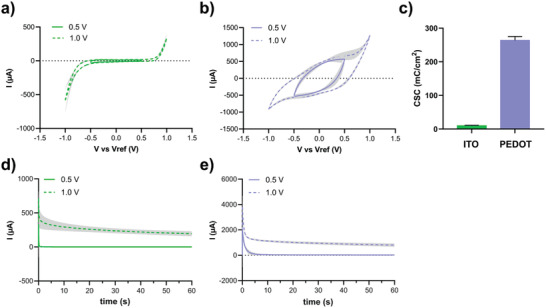
Electrochemical characterization showing charge storage in PEDOT:PSS/ITO slides. CV of a) ITO slides, and b) PEDOT:PSS/ITO slides performed within limits of ±0.5 V (solid lines) and ±1.0 V (dotted lines). The grey area behind the lines indicated standard deviation of all recorded cycles. c) Mean values and standard deviation of CSC calculated from cyclic voltammograms with *n* = 3. Chronoamperometry of d) ITO slides, and e) PEDOT:PSS/ITO slides performed at 0.5 V (solid lines) and 1.0 V (dotted lines). The grey area behind the lines indicated standard deviation of all recorded chronoamperograms (*n* = 3).

As the experimental setup of the study requires complete coating of a microscope slide with standard dimensions of 2.5 × 7.5 cm, we needed to ensure that the surface conductivity was sufficiently high. Therefore, we chose commercially available glass slides coated with ITO to serve as a highly conductive sublayer for the PEDOT:PSS. We performed electrochemical characterization of ITO and PEDOT:PSS/ITO slides using a standard three‐electrode setup with a liquid filled silver/silver chloride (Ag/AgCl) reference electrode and a Pt wire as counter electrode. As electrolyte, we used a salt free formulation of Luria‐Bertani broth (LB medium), which is a bacterial culturing medium known to promote *Salmonella* growth and biofilm formation. Cyclic voltammetry (CV) was performed with limits of ±0.5 and ±1.0 V to define charge storage capacities. Charge storage capacity (CSC) was calculated from the area under the curve (AUC) of voltammograms normalized with scan rate (0.1 V s^−1^) and electrochemical surface area (13.75 cm^2^) as described in Lipus et al.^[^ ^45^ ^]^ In the voltammograms of ITO with limits of ±0.5 V, anodic and cathodic current reaches a maximum of 12.5 µA indicating that little charge is transferred to the material. When the limits were increased to ±1.0 V, current flow in the capacitive part of the voltammogram hardly increased, but current peaks were observed in the faradic part of the voltammogram indicating the occurrence of consuming electrochemical processes. The average CSC was 972 ± 143 mC cm^−2^ when the potential was cycled within 0.5 V limits. Conversely, voltammograms of PEDOT:PSS/ITO slides showed the rounded rectangular shape typical of a pseudocapacitor when voltammograms were recorded with limits of ±0.5 V (**Figure** **2**b). Yet again, when limits were increased to ±1.0 V, current peaks appeared at the respective limit indicating occurrence of consuming electrochemical processes. When voltammograms were recorded with limits of ±0.5 V, the average CSC was 26 356 ± 1153 mC cm^−2^. The CSC for ITO and PEDOT:PSS/ITO are compared in Figure 2c.

To study the dynamics of charge transfer, i.e., the time it takes to fully polarize ITO slides and fully oxidize PEDOT:PSS/ITO slides, we performed chronoamperometry with anodic bias of 0.5 and 1.0 V. Current decay was fitted using a one‐phase exponential decay, and half‐life times and plateau values were determined for each condition. The current decay for ITO slides was very sharp, with half‐life time ≈0.2 s when biased with 0.5 V and 0.16 s when biased with 1.0 V (Figure 2d). Current values decreased below 1 µA when the material was biased with 0.5 V, and a plateau current of ≈250 µA was observed when the material was biased with 1.0 V. The latter indicated the presence of nonspecific electrochemical reactions such as oxidation of components of the LB medium and water electrolysis. The PEDOT:PSS/ITO slides showed a half‐life time of 1.6 s when biased with 0.5 V and 0.9 s when biased with 1.0 V (Figure 2e). The current decreased below 0.1 mA when the material was biased with 0.5 V, and a plateau current of more than 1 mA was observed when the material was biased with 1.0 V. Again, this indicated the presence of nonspecific electrochemical reactions when materials were biased at higher voltage. Taken together, our results showed that 0.5 V was ideal for extended addressing using LB medium as electrolyte, since the risk of overoxidation as well as water electrolysis was avoided at this bias potential. Further, we found a 22‐fold increase in charge storage capacity in PEDOT:PSS/ITO slides compared to ITO slides. With these parameters in mind, we defined the PEDOT:PSS/ITO slides as highly suitable for the ensuing biofilm experiments.

### 2.3 Biofilm Reactor Design for Electroactive Surfaces Reveals Bacteria‐Associated Cathodic Currents

For the aim of the present study, we had to develop a new type of biofilm reactor that fulfilled the following essential criteria: i) an autoclavable device allowing long‐term bacterial growth in liquid culture inside a 28 °C incubator; ii) the design should allow one or two electroactive surfaces to be positioned in the culturing device, on which surface interface biofilms can form at the air‐liquid interface; iii) the design must enable placement of electric leads for constant addressing of the electroactive surfaces and current acquisition without interfering with sterility; iv) the technique to withdraw the electroactive surfaces must clearly separate the pellicle from the surface interface biofilm, and v) the electroactive surfaces must be formatted to enable end‐stage analysis of the surface interface biofilm by spectroscopy and microscopy. We based our biofilm reactor on a standard glass Coplin staining jar, which normally is used when staining tissues slices mounted on glass slides prior to histological analysis. To the glass staining jar, we fitted a custom‐designed, autoclavable, 3D‐printed lid with holders for insulated banana plugs, allowing us to contact the electrodes and/or our electroactive slides without risk of contamination. The holders were kept in the same position for each experiment, and the filling volume was controlled such that variation of the electrochemical surface area was kept at a minimum. Once the biofilm reactor was filled with bacterial culture obtained from exponential phase liquid cultures in LB medium, the lid holding the electrodes was fitted and the biofilm reactor was positioned in a 28 °C bacterial incubator. The electric leads were connected to a Keithley 2602 sourcemeter placed atop the incubator and to a laptop, allowing control of potential and acquisition of current values in real‐time during biofilm formation.

**Figure 3:**
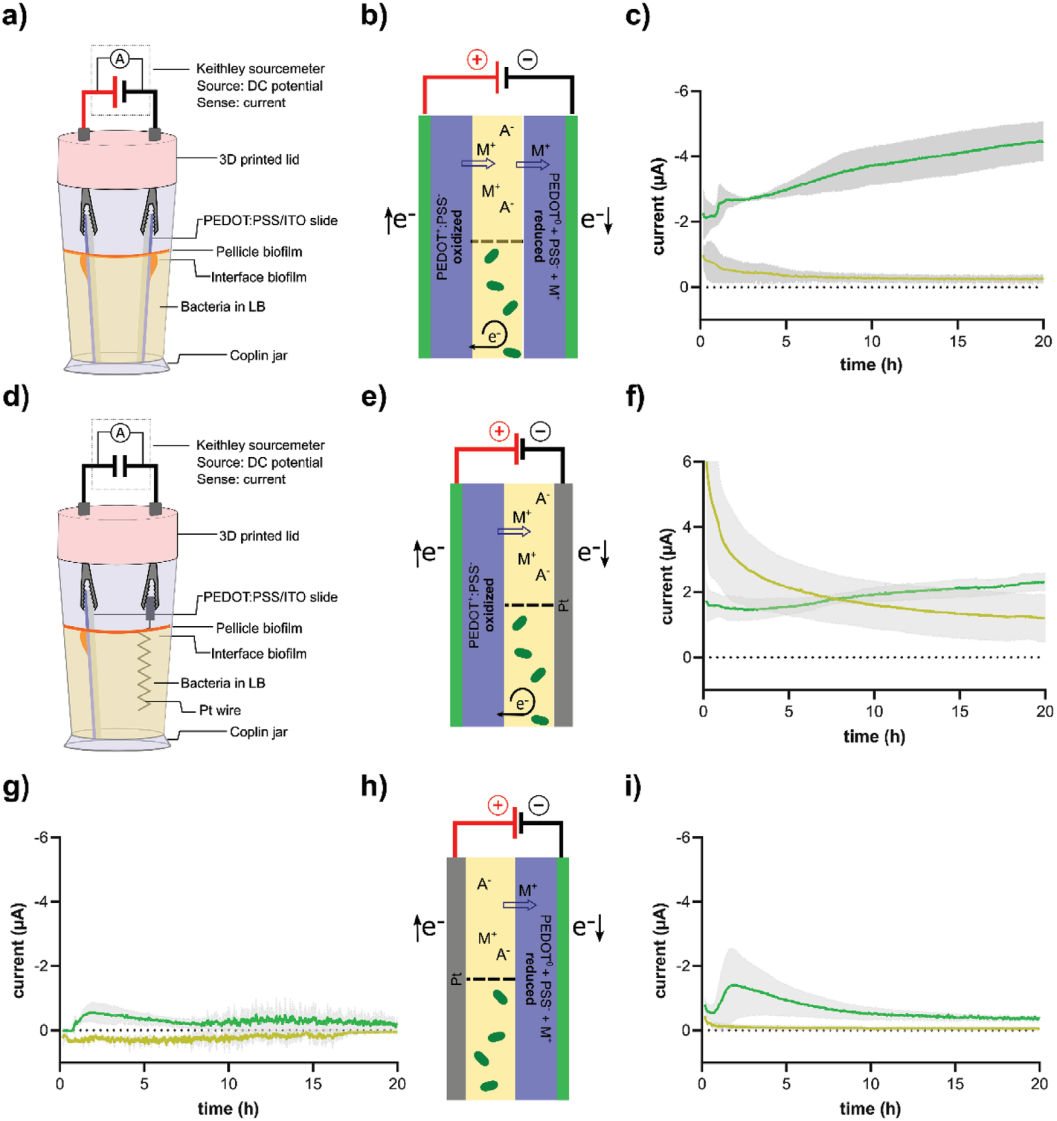
Biofilm reactor for air–liquid surface interface biofilm formation on PEDOT:PSS/ITO slides, the electrode setup enabling electrical addressing of surfaces, and resulting currents during *Salmonella* biofilm formation. a) Schematic of the biofilm reactor in whole‐cell mode, in which two vertically positioned PEDOT:PSS/ITO slides, used as anode and cathode, are electrically addressed during 20 h incubation of *Salmonella* wt‐GFP in LB medium at 28 °C. b) Cartoon illustrating electrochemical reactions in the whole‐cell setup using sterile LB medium (upper part), and a culture of *Salmonella* wt‐GFP (lower part). c) The current flowing though the electronic circuit in sterile LB medium (yellow lines) and in cultures of *Salmonella* wt‐GFP (green lines) at −0.5 V bias potential. d) The biofilm reactor in half‐cell mode, containing one PEDOT:PSS/ITO slide as working electrode and one Pt wire as counter electrode. e) Electrochemical half‐cell oxidization of PEDOT:PSS in sterile LB medium (upper part) and in a culture of *Salmonella* wt‐GFP (lower part). f) The anodic half‐cell current flowing though the electronic circuit in sterile LB medium (yellow lines) and in cultures of *Salmonella* wt‐GFP (green lines) at +0.5 V bias potential. g) Open‐circuit current, i.e., no potential applied (0 V), in sterile LB medium (yellow lines) and in cultures of *Salmonella* wt‐GFP (green lines). h) Electrochemical half‐cell reduction of PEDOT:PSS in sterile LB medium (upper part) and in a culture of *Salmonella* wt‐GFP (lower part). i) The half‐cell current flowing though the electronic circuit in sterile LB medium (yellow lines) and in cultures of *Salmonella* wt‐GFP (green lines) at −0.5 V bias potential. Dotted lines indicate zero current. The *y*‐axes in panel (c), (g), and (i) are reversed for improved visualization. Standard deviations based on 3–5 experimental repeats are shown in grey.

To study whether electroactive surface modulation affects biofilm physiology, we used a wt strain of *S*. Enteritidis genetically modified to express GFP.^[^ ^42^ ^]^ This strain (*Salmonella* wt‐GFP) is well‐known to form biofilm at air‐liquid interfaces when grown in a salt free formulation of LB medium at 28 °C. First, we used an electrochemical whole‐cell setup (**Figure** **3**a). This setup accommodated two PEDOT:PSS/ITO slides in the bacterial culture, and accordingly, this biofilm reactor design resulted in an electrochemical cell in which the PEDOT:PSS/ITO slide connected to the positive terminal was oxidized (PEDOT^+^) and produced an anodic current, and the slide connected to the negative terminal was reduced (PEDOT^0^), producing a cathodic current (Figure 3b).

We positioned the two PEDOT:PSS/ITO slides in LB medium inoculated with *Salmonella* wt‐GFP, applied a bias potential and simultaneously started to record the current. Biasing and recording were maintained over the following 20 h as the bacterial culture was incubated inside a 28 °C incubator. Initially, a sharp increase followed by a rapid decay in net current was observed. Since these changes are related to electrochemical oxidation and reduction processes in the material and formation of electrochemical double layers, we only showed current data obtained from 10 min of incubation and onward (Figure 3c). After circa 45 min, a gradual increase in net current was observed. Starting at circa 2 µA, the net current increased due to the growth of the bacterial culture, reaching 4.5 µA after 20 h incubation. This confirms our previous finding, showing that bacterially produced metabolites contribute to reduction of PEDOT:PSS, producing a small but continuous flow of current throughout the experiment.^[^ ^13^, ^46^ ^]^ Using sterile LB control medium, a slight residual current related to completion of electrochemical charging of the material was observed. The current eventually decreased to ≈0.2 µA, which we considered as the leakage current of the electrochemical cell. Whereas a great advantage of the whole‐cell setup is that the open circuit potential is around zero since both electrodes are of the same material, a drawback is that the role of anodic or cathodic components cannot be analyzed individually.

To be able to dissect anodic or cathodic currents, we developed a half‐cell setup (Figure 3d). To control the potential at the PEDOT:PSS/ITO slide in the half‐cell mode, we used an inert Pt electrode as counter electrode. We refrained from using the traditional 3‐electrode setup that includes a Ag/AgCl reference electrode, since Ag and Ag^+^ exert anti‐bacterial and anti‐biofilm effects^[^ ^36^ ^]^ and present problems related to clogging of the liquid junction in liquid filled electrodes. In our half‐cell setup, we applied a positive potential to the PEDOT:PSS/ITO slide to observe PEDOT:PSS oxidation (PEDOT^0^ → PEDOT^+^ + e^−^) and electrochemical processes related to bacterial growth (Figure 3e). As we recorded the anodic half‐cell current in the *Salmonella* wt‐GFP culture, we found the current to decline to a minimum of 1.4 µA after 2.5 h (Figure 3f). From this point onward, the current increased, reaching 2.4 µA at the end of incubation. In the absence of bacteria, slow decline of the anodic current was observed throughout the full length of the experiment. This suggested that in our half‐cell setup, the actual potential between the PEDOT:PSS/ITO slide and the Pt wire differs from the applied potential, and that PEDOT:PSS undergoes slow oxidation throughout the entire time of incubation. In the presence of bacteria, this effect was most likely opposed by bacterially produced metabolites, which reduce the material. We next analyzed the response of a PEDOT:PSS/ITO slide which we kept at open circuit potential (unbiased) during bacterial growth. With no electrical addressing, this experiment was designed to show whether bacterial growth influences the redox state of PEDOT:PSS. In the *Salmonella* wt‐GFP culture, a slight increase in net current was observed after 30 min incubation, reaching a peak of 0.6 µA at 2 h (Figure 3g). In the sterile LB control medium, the current remained around zero throughout the experiment. Collectively, data shown in Figure 3 illustrate that bacterial growth contributes to net current increase when the material is continuously regenerated by an oxidating electrical current. This corroborates our previous finding, which showed the net current increase to originate from reductive extracellular metabolites produced by bacteria.^[^ ^16^ ^]^

We then recorded the cathodic half‐cell current in the *Salmonella* wt‐GFP culture by applying a negative potential such that the PEDOT:PSS/ITO slide was reduced (PEDOT^+^ + e^−^ → PEDOT^0^) (Figure 3h). In the sterile LB control medium, the cathodic net current decayed quickly and remained zero throughout the experiment (Figure 3i). This indicated that a complete reduction of PEDOT:PSS was maintained throughout the experiment. Interestingly, in the presence of bacteria, a peak in net current at ≈2 h was observed. This pattern mimicked that of the unbiased case, although a higher maximum was reached in the reduced case. Since PEDOT:PSS became fully reduced by electric addressing, as shown in the sterile LB control medium experiment, we concluded that the peak at 2 h represented a secondary, bacteria‐associated process that fed electrons into the circuit. Taken together, by dissecting the anodic or cathodic currents in our half‐cell setup, we found that in addition to PEDOT reduction, other bacteria‐related processes also lead to reduction. From a bacterial physiology perspective, it is interesting to notice that the net current showed a peak at ≈2 h rather than constant increase. This indicated that bacteria‐associated reduction only occurred during a given growth phase.

### 2.4 Biofilm Evaluation at the Macroscopic Scale Reveals Independent Regulation of ECM

Biofilms represent highly regulated consortia of bacterial cells. In addition to cell proliferation and multiplication, bacteria engage in the production of ECM components. Biofilm formation is thus strictly regulated by bacterial gene expression at multiple levels. This suggests that the redox‐induced changes we observed in the whole‐cell and half‐cell setups may either influence the growth of bacterial cells, and/or they may act on gene expression regulating the production of ECM components. To analyze this, we generated macroscopic fluorescence intensity maps of surface interface biofilm formed on PEDOT:PSS/ITO slide(s) at the air‐liquid interface. The procedure started by growing *Salmonella* wt‐GFP for 20 h in LB medium at 28 °C within the whole‐cell and half‐cell setups, in the presence of PEDOT:PSS/ITO slide(s) to which we applied a bias potential of 0.5 or 1.0 V while recording the current. At the end of incubation, we cautiously pulled out the slides on which the surface interface biofilm remained attached without contamination from the pellicle. After the backside of the slides were wiped clean, we stained the ECM of the surface interface biofilm by addition of EbbaBiolight 680. To quantify the fluorescence from bacterial cells based on GFP expression and ECM based on EbbaBiolight 680 fluorescence, we mounted the slides on a 4‐slide holder fitted to the standard dimension of microwell‐plates used in spectrophotometric plate readers. Recording of fluorescence from the entire area of surface interface biofilms was achieved by programming the spectrophotometer such that the entire slide area was mapped with a pixel dimension corresponding to 1.3 mm x 1.3 mm. Using single wavelength detection, we recorded the fluorescence intensity of GFP and EbbaBiolight 680, and generated colormaps in which each pixel was color‐coded according to its fluorescence intensity. We quantified the amounts of cells and ECM by averaging the fluorescence intensity in each row and calculating the AUC of the resultant vertical line profile.

**Figure 4:**
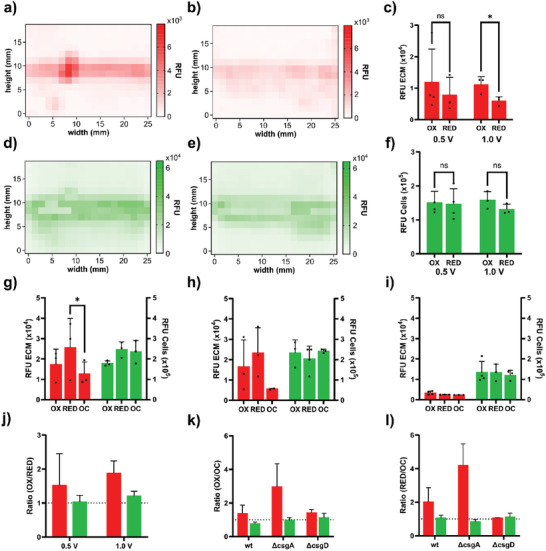
Spectroscopic mapping for individual quantification of bacterial cells and ECM in surface interface biofilms formed on electroactive surfaces. The averaged results for spectroscopic mapping shown as colormaps, in which the intensity (RFU) of each pixel is color coded. a,b) Colormaps of *Salmonella* wt‐GFP biofilm formed on PEDOT:PSS/ITO slides in whole‐cell setup at 0.5 V bias potential labeled with EbbaBiolight 680 (red) showing the average fluorescence intensity (RFU) on a) oxidized material, and b) reduced material. c) Quantification of colormaps shown in (a,b). Data for 1.0 V bias potential of oxidized surfaces originates from colormaps shown in Figure S2a,b (Supporting Information). d,e) Colormaps of *Salmonella* wt‐GFP biofilm on PEDOT:PSS/ITO slides in the whole‐cell setup at 0.5 V bias potential showing the RFU of GFP (green) representing bacterial cells on d) oxidized material, and e) reduced material. f) Quantification of colormaps shown in (d,e). Data for 1.0 V bias potential of reduced surfaces originates from colormaps shown in Figure S2c,d (Supporting Information). g–i) Quantification of colormaps of biofilms formed by g) *Salmonella* wt‐GFP, (h) *Salmonella* Δ*csgA*‐GFP, and i) *Salmonella* Δ*csgD*‐GFP on oxidized (0.5 V), reduced (0.5 V), and unbiased (OC) PEDOT:PSS/ITO slides in the half‐cell setup. Colormaps for each of the strains are shown in Figures S3–S5 (Supporting Information), respectively. j) The ratio of EbbaBiolight 680 (red) representing the ECM, and GFP (green) representing bacterial cells on oxidized versus reduced PEDOT:PSS/ITO slides biased at 0.5 and 1.0 V. k) The ratio of EbbaBiolight 680 (red) representing the ECM, and GFP (green) representing bacterial cells in biofilms formed by *Salmonella* wt‐GFP (wt), *Salmonella* Δ*csgA*‐GFP (Δ*csgA*), and *Salmonella* Δ*csgD*‐GFP (Δ*csgD*) on oxidized versus unbiased PEDOT:PSS/ITO slides in the half‐cell setup. l) The ratio of EbbaBiolight 680 (red) representing the ECM, and GFP (green) representing bacterial cells in biofilms formed by *Salmonella* wt‐GFP (wt), *Salmonella* Δ*csgA*‐GFP (Δ*csgA*), and *Salmonella* Δ*csgD*‐GFP (Δ*csgD*) on reduced versus unbiased PEDOT:PSS/ITO slides in the half‐cell setup. Standard deviations (c,f,g,h,i) and standard errors (j,k,l) are shown for 3 experimental repeats per experiment.

First, we generated macroscopic fluorescence intensity maps of surface interface biofilms formed at the air‐liquid interface of biased PEDOT:PSS/ITO slides in the whole‐cell setup. At 0.5 V bias potential, an averaged color‐coded fluorescence intensity map was generated for ECM when the material was oxidized (**Figure** **4**a) or reduced (Figure 4b). Interestingly, the interface biofilms were observed as sharp bands located at the air‐liquid interface on the oxidized surface and fainter on the reduced surface. Quantification of the relative fluorescence of ECM (red) revealed high variability amongst the experimental repeats (Figure 4c). As we performed the experiment using 1.0 V bias potential, the averaged colormap (Figure S2a,b, Supporting Information) and associated quantification (Figure 4c) showed the same trend as 0.5 V, but with much less variability. Multiple unpaired t‐test showed no significance at 0.5 V (p = 0.58), whereas the difference at 1.0 V were significant (p = 0.038).

As we generated averaged colormaps representing relative fluorescence units (RFU) from bacterial cells expressing GFP (Figure 4d,e), quantification revealed high variability of data at 0.5 V bias potential (Figure 4f). In contrast, data from the averaged colormaps obtained from 1.0 V bias (Figure S2c,d, Supporting Information) showed much less variability (Figure 4f). At both bias potentials, comparison of the RFU from cells on oxidized and reduced materials by multiple unpaired t‐test showed no significant differences.

To analyze if the ECM increase is related to production of curli and/or cellulose, known as main constituents of *Salmonella* ECM, we introduced two GFP‐expressing mutants. *S*. Enteritidis Δ*csgA* (*Salmonella* Δ*csgA*‐GFP) which is unable to express curli, and *S*. Enteritidis Δ*csgD* (*Salmonella* Δ*csgD*‐GFP), which neither express curli nor cellulose and is therefore a very poor biofilm former. Using the half‐cell setup in which the PEDOT:PSS/ITO slides were biased at ±0.5 V or kept at open circuit potential, we grew cultures of *Salmonella* wt‐GFP, *Salmonella* Δ*csgA*‐GFP, and *Salmonella* Δ*csgD*‐GFP. Following 20 h incubation, we performed spectrophotometric analysis of the slides and generated averaged colormaps of surface interface biofilms formed at the air–liquid interface (Figures S3–S5, Supporting Information). Quantification of the fluorescence intensity of ECM (red) and bacterial cells (green) for *Salmonella* wt‐GFP (Figure 4g), *Salmonella* Δ*csgA*‐GFP (Figure 4h), and *Salmonella* Δ*csgD*‐GFP (Figure 4i) was performed. When comparing the amount of bacterial cells on the oxidized, reduced and unbiased PEDOT:PSS/ITO slides, we found *Salmonella* Δ*csgA*‐GFP to show the same relative amount (Figure 3h). This was expected since the mutation does not affect bacterial growth. However, the apparent ECM signals on the charged surfaces were unexpected, since the mutation in Δ*csgA* is known to abrogate bacterial production of curli. In the *Salmonella* Δ*csgD*‐GFP mutant, which is unable to produce curli as well as cellulose, the ECM signal was barely detectable and the signal for bacterial cells was markedly decreased (Figure 3i). This confirms this mutants’ inability to form biofilm. Also, the data confirms that under present conditions, EbbaBiolight 680 detects the ECM polysaccharide cellulose in addition to curli within the biofilm.

As we performed statistical testing with a one‐way ANOVA analysis with an uncorrected Dunn's test for multiple comparison, the only case showing a significant difference was between the quantities of ECM on reduced compared to open circuit surfaces (p = 0.041) in *Salmonella* wt‐GFP (Figure 4g). We consistently observed increased quantities of ECM on addressed surfaces compared to non‐addressed surfaces. Therefore, we performed ratio determination by comparing the amount of ECM and bacterial cells on oxidized, reduced, and unbiased surfaces. When analyzing ECM and bacterial cells on oxidized compared to reduced surfaces in the whole‐cell setup at 0.5 and 1.0 V, we found in both cases a relative increase of ECM the oxidized surfaces, while the relative amount of bacterial cell remained unchanged (Figure 4j). When performing ratio analysis with mutant strains in the half‐cell setup, we used the unbiased surface as comparator. The relative amount of ECM is increased on oxidized compared to unbiased surfaces in all strains, but the relative amounts of cells remains unchanged or slightly decreased (Figure 4k). Again, this illustrates that electric biasing of the surfaces has greater influence on bacterial ECM production than on the actual cell numbers. Also, this finding shows that electrically biasing influences curli aggregation as well as the amount of cellulose.

### 2.5 High‐Resolution, Large Area Image Analysis Reveals Biofilm Component Distribution on Functionalized Surfaces

#### 2.5.1 Imaging and Image Processing to Achieve Microscopic‐to‐Macroscopic Scale Transition

To gain information on the differential distribution of bacterial cells and ECM components in surface interface biofilms formed at the air–liquid interface, we developed a protocol that combined the detailed picture obtained by confocal laser scanning microscopy and large area analysis. To overcome the limitation of small area observation offered by standard objectives (10‐63 X magnification) on the confocal microscope, we visualized our biofilms by acquiring fluorescence images of GFP‐expressing bacteria (green) and EbbaBiolight 680 stained ECM (red) in the form of 5 rows and 2 columns, generating a 5 × 2 tiled grid. The 5 × 2 tiles were stitched together to generate a complete image of the focal plane. This process was repeated for each focal plane obtained at z‐steps of 1.98 µm, resulting in a 3D image matrix. By summing the intensities in the z direction, we generated a z‐projection representing a total area of 1.2 cm x 2.9 cm (width x height) at ≈40 µm total biofilm thickness. By merging the green and red channels, we obtained a color composite image of the total surface interface biofilm. This high‐resolution, large area image analysis enabled us to show the distribution of bacterial cells and ECM throughout the entire surface interface biofilm.

In addition to visualizing the biofilm, we wanted to quantify the distribution of cells and ECM. We therefore analyzed the two color channels individually using their respective z‐projections. The RFU values were sorted into 256 bins (flexible bin‐size) and the number of RFUs in a certain bin (counts) was calculated. To obtain an average histogram representing 3–5 experimental repeats, we aligned each histogram such that all maximum values were at zero bin number, then normalized the count values with respect to the maximum count. As the number of dark pixels in each image was quite high and lacked relevant information, we only plotted results from bins 22–256. The resulting histograms showed the tonal distribution of bacterial cells and ECM in the biofilms. From these histograms, we calculated the entropy, which represents the degree of randomness in the image. We calculated the entropy H = sum (p_i_ x log_2_p_i_) using probabilities p_i_ for the counts in each bin (count in bin i/sum of counts in all bins).

Also, we developed image processing to enable objective determination of the biofilm thickness. Biofilm thicknesses based on bacterial cells as well as ECM were analyzed using data from the 3D image matrix, studying the two‐color channels individually. For each focal plane, we defined the total intensity as the sum of RFUs in the plane. Also, we defined the distance of each plane from the bottom of the z‐stack by multiplying the plane number by the z‐step (1.98 µm). We then plotted the total intensity as a function of the distance from the bottom of the stack, to generate a RFU‐distance plot. To obtain an average RFU‐distance plot representing 3–5 experimental repeats, we aligned the maximum values of each plot such that the center of the biofilm was defined as 0 µm, and we normalized the RFU values with respect to the maximum RFU value. The resulting RFU‐distance plot showed the distribution of the biofilm material (bacterial cells and ECM) along the *z*‐axis, and the thickness was defined as the full‐width at half maximum (FWHM) intensity.

#### 2.5.2 Distribution Analysis of Bacterial Cells and ECM in Large Area Biofilms on Electroactive Materials

**Figure 5:**
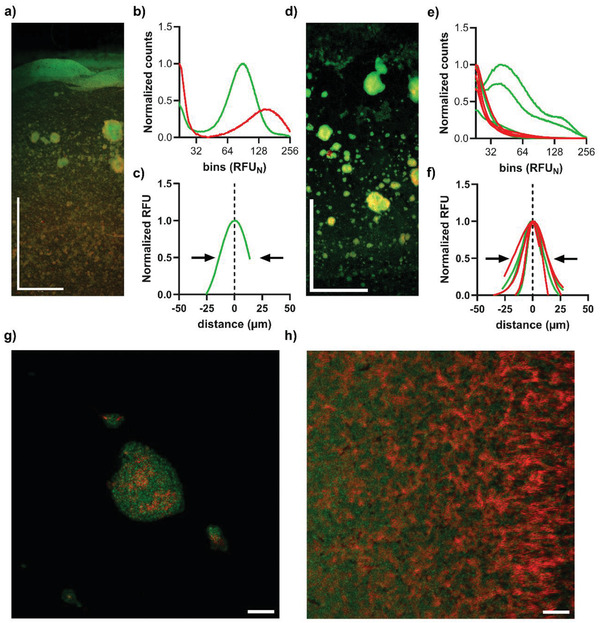
Distribution of ECM and bacterial cells in surface interface biofilms formed on glass and unbiased PEDOT:PSS/ITO. a) Tiled image stacks, acquired with confocal laser scanning microscopy, visualizing the distribution of ECM (red) and bacterial cells (green) in surface interface biofilm grown on glass. Scale bars: horizontal 0.5 mm, vertical 1.0 mm. (*n* = 1) b) Histograms showing the fluorescence intensity distribution of EbbaBiolight 680 (red) representing the ECM, and GFP (green) representing bacterial cells in biofilms formed on glass. (*n* = 1) c) The total intensity for EbbaBiolight 680 (red) representing the ECM, and GFP (green) representing bacterial cells shown as normalized RFU for each slice plotted against the distance from the maximum. FWHM is indicated by arrows. d–f) Analysis of biofilms formed on unbiased PEDOT:PSS/ITO slides using the same principles as in (a–c). d) Tiled image stacks visualizing the distribution of ECM (red) and bacterial cells (green) in surface interface biofilm grown on glass. Scale bars: horizontal 0.5 mm, vertical 1.0 mm. e) Histograms showing the fluorescence intensity distribution of EbbaBiolight 680 (red) representing the ECM, and GFP (green) representing bacterial cells in biofilms formed on unbiased PEDOT:PSS/ITO slides. f) The total intensity for EbbaBiolight 680 (red) and GFP (green) shown as normalized RFU for each slice plotted against the distance from the maximum. FWHM is indicated by arrows. g,h) High magnification (63x) confocal microscopy image of g) a single, small foci consisting of a cluster of bacterial cells (green) with interspersed ECM (red) between cells on unbiased PEDOT:PSS/ITO, and h) a region showing a more continuous structure of the biofilm on unbiased PEDOT:PSS/ITO, with bacterial cells (green) and an increasing amount of ECM (red) toward the periphery. Scale bar = 10 µm. All experiments with PEDOT:PSS/ITO slides were performed with *n* > 3. Due to the nature of the imaging analysis, not each experiment provided the full amount of information. Green and red lines indicate individual repeats obtained for each analysis.

To develop the above‐mentioned image processing protocol allowing detailed confocal microscopy‐based analysis over large areas of the biofilm, we started by comparing *Salmonella* wt‐GFP biofilm formation on glass slides (rigid surface) and unbiased PEDOT:PSS/ITO slides (softer surface). As we performed large area image analysis of biofilms formed on glass, a cell‐rich layer facing toward the direction of air was observed (**Figure** **5**a). An intermediate layer featured foci of attached cells and ECM. Further down, toward the bulk of the liquid, a homogeneous and carpet‐like biofilm had formed with evenly distributed cells surrounded by ECM. This distribution was also reflected in the histogram (Figure 5b). Here, the upper cell‐rich layer and the interface foci were represented by GFP (green line) fluorescence in high‐intensity bins 48–128, and a peak centered at the 96th bin. The lower, evenly distributed biofilm region was represented by the low‐intensity region of the histogram, showing a shoulder at bin 22–30. When analyzing the histogram for EbbaBiolight 680 (red line) fluorescence, we found a high intensity region at bins 96–256 and low intensity region at bins 22–32. Thus, the distribution of ECM differed from that of bacterial cells. In the high intensity region, the relative counts of GFP were higher than EbbaBiolight 680, while the opposite was observed in the lower intensity region. This suggests that ECM is differentially expressed in different regions of the biofilm. The distribution of biofilm material along the *z*‐axis is shown in Figure 5c. When studying surface interface biofilms grown on unbiased PEDOT:PSS/ITO slides, our high‐resolution, large area image analysis revealed a biofilm with little similarity to the carpet‐like structure formed on glass (Figure 5d). Cell‐rich foci had formed toward the air. The intermediate layer was less pronounced, instead numerous foci containing bacterial cells and ECM were observed. Further down, toward the bulk of the liquid, foci were smaller and more evenly distributed, but no carpet‐like structure had formed. These observations were also reflected in the histograms, in which the high‐intensity GFP fluorescence observed on glass now had shifted to bins 32–96, centered at the 48th bin (Figure 5e). A shoulder was occasionally observed at bin 128, indicating cell‐rich, high‐intensity foci in localized regions. Interestingly, the high‐intensity peaks from EbbaBiolight 680 seen on glass slides were shifted to lower intensities in biofilms grown on the PEDOT:PSS/ITO slides. This provides evidence that PEDOT:PSS already in unbiased state affects cellular processes leading to ECM production. The distribution of biofilm material along the *z*‐axis is shown in Figure 5f. To illustrate the different structures and distribution of bacterial cells and ECM on unbiased PEDOT:PSS/ITO slides, a high magnification, confocal microscopy image of a single, small foci consisting of a cluster of bacterial cells (green) with interspersed ECM (red) between cells is shown (Figure 5g). In other areas, bacterial cells (green) showed a more continuous distribution, in which an increasing amount of ECM (red) was produced at the periphery (Figure 5h).

**Figure 6:**
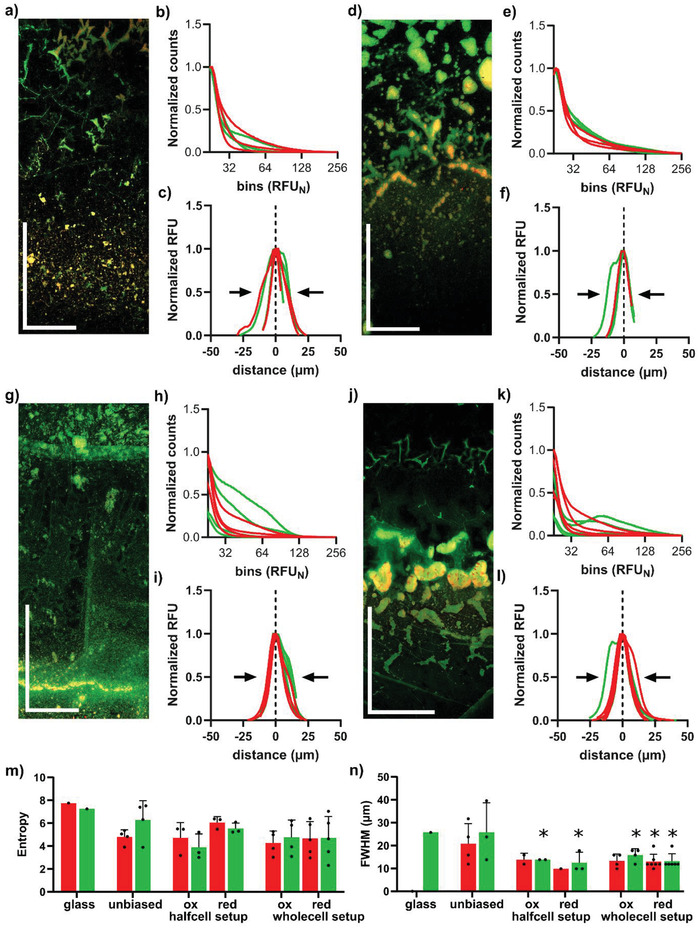
Distribution of ECM and bacterial cells in surface interface biofilm grown on electrically addressed PEDOT:PSS. Analysis of biofilms formed by Salmonella wt‐GFP on oxidized and reduced PEDOT:PSS/ITO surfaces addressed with 0.5 V bias potential in (a–f) half‐cell and g–l) whole‐cell setups. Tiled image stacks visualizing the distribution of ECM (red) and bacterial cells (green) grown on (a,g) oxidized and d,j) reduced slides in the (a,d) half‐cell and g,j) whole‐cell setup. Scale bars: horizontal 0.5 mm, vertical 1.0 mm. Histograms showing the fluorescence intensity distribution of EbbaBiolight 680 (red) representing the ECM, and GFP (green) representing bacterial cells in biofilms formed on (b,h) oxidized and e,k) reduced slides in (b,e) half‐cell and h,k) whole‐cell setups. Total fluorescence intensity for EbbaBiolight 680 (red) representing the ECM, and GFP (green) representing bacterial cells shown as normalized RFU for each slice plotted against the distance from the maximum in biofilms formed on (c,i) oxidized and f,l) reduced surfaces in (c,f) half‐cell and i,l) whole‐cell setups. Each red and green line represents an individual experiment. *n* > 3 for all experiments. Due to the nature of the imaging analysis, not each experiment provided the full amount of information. FWHM is indicated by arrows. m) Entropy of the probability distribution of RFU values for EbbaBiolight 680 (red) representing the ECM, and GFP (green) representing bacterial cells calculated from histograms shown for glass (Figure 5b), unbiased PEDOT:PSS/ITO slides (Figure 5e), as well as oxidized (ox) and reduced (red) surfaces from half‐cell (Figure 6b, e) and whole‐cell (Figure 6h,k) setups. n) Summary of the thickness (FWHM) of the biofilms, obtained from data presented in Figures 5c,f and 6c,f,i,l. Comparison of entropy and FWHM on unbiased and electrically addressed surfaces was achieved by mixed‐effects analysis with uncorrected Fisher's test. Significant changes are indicated by ^*^.

Next we analyzed if electrical addressing of the PEDOT:PSS/ITO slides exert any effects on biofilm growth. Following incubation in the half‐cell setup biased with ±0.5 V, we performed high‐resolution, large area image analysis of biofilms formed on the oxidized and reduced surfaces. Biofilm formed on the oxidized surface showed an irregular upper region rich in cells and ECM, whereas the lower region showed a uniform distribution of cells and ECM (**Figure** **6**a). Histograms showed that the intensity counts from both GFP and EbbaBiolight 680 were skewed toward the low‐intensity bins 22–64 (Figure 6b). This pattern differed from that observed in the unbiased situation. The distribution of biofilm material along the *z*‐axis is shown in Figure 6c. On reduced surfaces, the biofilm showed high‐intensity, large‐sized foci of bacterial cells and ECM (Figure 6d). The histograms showed that intensity counts originating from both bacterial cells and ECM were skewed toward low‐intensity bins 22–64 (Figure 6e). This was similar to what was observed for the oxidized surface. The distribution of biofilm material along the *z*‐axis is shown in Figure 6f.

We then performed experiments using the whole‐cell setup biased with 0.5 V. High‐resolution, large area image analysis of biofilms formed on the oxidized surface revealed two cell‐rich layers (Figure 6g). The upper dense layer was separated, by a low cell‐density gap, from a lower cell‐rich layer. The ECM was predominantly observed in the lower layer where it colocalized with bacterial cells. The histograms showed the EbbaBiolight 680 intensity distribution to be skewed toward low‐intensity regions (bins 22–64), while GFP intensity was distributed over a wider intensity range (bins 22–128) (Figure 6h). The distribution of biofilm material along the *z*‐axis is shown in Figure 6i. On the reduced surface, the biofilm showed a faint upper region containing bacterial cells, as well as a lower region containing bacterial cells and a high signal for ECM (Figure 6j). The foci were much more prominent and covered a larger area compared to what was observed on the oxidized and the unbiased surfaces. This pattern seemed to be characteristic for reduced surfaces, irrespective of setup. In the histograms, GFP intensity counts showed a peak between bins 32 – 128, and EbbaBiolight 680 intensity distribution was skewed to lower intensities (Figure 6k). The distribution of biofilm material along the z axis is shown in Figure 6l.

To objectively characterize the texture of the biofilms, we calculated the entropy for each biofilm at each condition (Figure 6m). The entropy values for both GFP and EbbaBiolight 680 were highest on glass. This reflected a high degree of randomness in the image, likely linked to the carpet‐like structure of biofilms only observed on glass surfaces. On unbiased PEDOT:PSS/ITO surfaces, lower entropy values were observed, reflecting the foci‐rich organization of the biofilms. When biofilms were grown on surfaces with incomplete oxidation and reduction in the half‐cell setup, the trend toward lowered entropy values continued. Biofilms grown on completely oxidized and reduced surfaces in the whole‐cell setup showed the lowest entropy values. Low entropy is indicative of a low degree of randomness in the biofilm distribution, related to the localization of biofilm within small and large foci.

To analyze the thickness of the biofilms, we used the FWHM from the RFU‐distance plots of each condition (Figures 5c,f and 6c,f,i,l), in which horizontal arrows indicated the thickness. A summary of FWHM values for all conditions is shown in Figure 6n. On glass, the FWHM was 25.7 µm, determined from GFP fluorescence of the bacterial cells. Unbiased PEDOT:PSS/ITO surfaces showed FWHM of 20.8 µm for ECM and 25.7 µm for GFP. Biofilms growing on biased PEDOT:PSS/ITO surfaces showed a thickness ranging between 9.9 and 15.8 µm. Supported by the statistical analysis (see Figure 6n), we conclude that glass and unbiased PEDOT:PSS/ITO supported formation of thicker biofilms compared to the electrically addressed surfaces. When compiling information gained from our high‐resolution, large area image analysis, our quantification of the distribution of cells and ECM within the biofilms, as well as the biofilm thickness analysis, we conclude that the spatial distributions of bacterial cells and ECM are affected by the bias, i.e., whether surfaces are reduced or oxidized, as well as the dynamics of surface charging.

## 3 Discussion

Here, we have presented numerous methodological improvements allowing us to analyze if electronic addressing of electroactive surfaces can influence bacterial biofilm formation, and if so, whether such an effect targets bacterial cell attachment and multiplication and/or the production of ECM. First, we constructed a biofilm reactor supporting surface interface biofilm formation at air–liquid interfaces. Our design is based on vertical positioning of standard microscopy slides in a glass Coplin jar, which allows for testing of a versatile range of materials given that the material under study can be coated onto the microscopy glass slide. Moreover, the slide format is ideal for spectroscopic and microscopic analysis once withdrawn at the end of incubation. To specifically analyze surface interface biofilm, the pellicle, i.e., the biofilm formed at the air‐liquid interface floating atop of the medium, must be prevented from fouling the surface biofilm formed on the slide. In our biofilm reactor, this is achieved by positioning the slides such that they face the wall of the Coplin jar. This ensured pellicle adhesion to the glass walls of the jar as functionalized slides were withdrawn for downstream analysis. To enable electronic addressing of electroactive surfaces inside the biofilm reactor, we designed a custom‐made 3D printed lid. This lid enabled addressing of the functionalized slides without compromising sterility. Also, the format of the Coplin jar provided the stability needed to enable recording of the current from the electrochemical cell during the entire length of bacterial incubation. Our biofilm reactor may find general use, since it can harbor slides with any type of functionalized coating.

Bacteria cultivated in the biofilm reactor experience different microenvironments at different locations of the reactor. Each microenvironment can dramatically influence the bacterial physiology, one example being bacterial growth at different oxygen concentrations. Our use of standard‐sized microscope slides positioned vertically in the growth medium ensured our assay to include different microenvironments, such as the upper area facing toward the air and lower area facing into the bulk of the liquid medium. To successfully coat the large area of a microscope slide (2.5 cm x 7.5 cm) with a layer of high charge storage PEDOT:PSS, we developed a protocol by which PEDOT:PSS was spin‐coated onto ITO coated glass slides of microscope slide dimensions. This large, conductive area was stable for long‐term incubation (>20 h) in aqueous solutions of bacterial growth medium, and electrochemical analysis confirmed that the high conductivity of the underlying ITO surface enabled successful addressing of the large PEDOT:PSS surface area. This was shown by the typical high charge storage capacity of PEDOT:PSS and characteristic shape of the cyclic voltammogram of PEDOT:PSS coated on ITO.^[^ ^47^, ^48^ ^]^

When analyzing the electrochemical responses of the electroactive material associated with biofilm formation within the biofilm reactor in whole‐cell and half‐cell mode, we found a reducing current produced by bacteria to contribute to PEDOT:PSS reduction. Also, growth of bacterial cells was feeding electrons into the circuit when PEDOT was fully reduced. We have previously reported that small, soluble metabolites produced by *Salmonella* and other clinically relevant bacteria lead to reduction of PEDOT, as detected using screen‐printed bioelectronic devices.^[^ ^16^ ^]^ Deposition of electrons into the material via other mechanisms, such as bacterial cells in direct contact to the electrode or via bacterial nanowires,^[^ ^49^, ^50^, ^51^ ^]^ can, however, not be excluded. We envision that continuous monitoring of current flow, as demonstrated here, will eventually be used as a tool to monitor bacterial growth and biofilm formation on electroactive surfaces.^[^ ^52^, ^53^, ^54^, ^55^ ^]^

The biofilm composition is very complex. Here, we applied fluorescence‐based techniques allowing us to separate signals of different components constituting a biofilms’ total biomass. Use of bacterial strains genetically engineered to express GFP allowed us to analyze the contribution of bacterial cells to the total biofilm, whereas specific detection of ECM was obtained by the fluorescent tracer molecule EbbaBiolight 680. Fluorescence signals from GFP and EbbaBiolight 680 are easily quantified and visualized by spectroscopy and microscopy. Further dissection of the effects of ECM components was achieved as we applied this technique to isogenic bacterial mutants lacking the expression of one or several ECM biomacromolecules. By establishing this fluorescence‐based method, we take several measures to surpass the present standards of the field. For decades, the most widely used method to quantify overall biomass of biofilm has been the Crystal violet assay. This dye is, however, not suitable for in situ biofilm imaging. Also, it binds a wide range of negatively charged polymeric substances, hindering detection of specific biofilm components. Binding to polar polymer materials also limits the use of this dye in experiments involving polymeric surface material. In contrast, we foresee that our method would be highly compatible with recent fluorescent stains used in biofilm research, such as DNA stains, membrane dyes, and matrix stains targeting proteins, given that the proper optical settings can be applied.^[^ ^56^, ^57^, ^58^, ^59^, ^60^, ^61^, ^62^, ^63^, ^64^ ^]^

When analyzing the behavior of biofilms on electroactive surfaces, our quantification of fluorescence intensities by spectroscopic mapping showed the amount of ECM to increase upon electroactive addressing, whereas the amounts of bacterial cells remained almost unchanged. In contrast, high‐resolution, large area image analysis showed that bacterial cells also reacted to electrical addressing of the material. Rather than viewing these data as contradictory, we believe they illustrate the importance of using complementary recordings, taking both the amount and distribution of bacterial cells and ÉCM into account. Such combined information is needed to enable better understanding of the biofilm organization and its reaction to stressors, including electrical addressing of the surfaces. Our large‐area image analysis showed biofilms to adopt different structures depending on surface conditions, as exemplified by its organization into differently sized foci at some surface conditions and into carpet‐like structures at others. Such changes at the macro‐scale would have gone unnoticed if high‐magnification imaging had been used exclusively. Collectively, this demonstrates the need for imaging and image processing that enables microscopic‐to‐macroscopic scale transition.

From a gene regulation perspective, it is notable that the differential influence of electronic addressing on the amount of ECM and bacterial cells was not limited to the curli‐expressing wt strain, the same pattern was seen for the curli mutant *Salmonella* Δ*csgA*‐GFP. This suggests that electroactive addressing may affect ECM regulation via a master regulator. The master regulator CsgD is a known responder to a variety of environmental stressors, thereby regulating expression of curli fimbriae as well as matrix polysaccharides.^[^ ^65^, ^66^, ^67^ ^]^ Here, we present the first evidence showing that electroactive addressing of surfaces also activates CsgD. Interestingly, CsgD expression has been shown to increase in the presence of copper chloride.^[^ ^66^ ^]^ As copper ions may act as electron acceptors, similar to what we recently reported for PEDOT surfaces,^[^ ^13^, ^16^ ^]^ we will in future studies investigate the regulatory pathways in *Salmonella* leading to increased ECM expression, including a potential role of the important CsgD regulator.

## 4 Conclusion

The fluorescence‐based method we present surpasses the present standards in the field of biofilm analysis. Our straightforward analysis workflow provides a holistic picture of the microscopic‐to‐macroscopic scale transition bacteria undergo when organizing themselves into the macroscopic communities significant of biofilms. The presented workflows allow for visualization, identification, and quantification of bacterial cells and ECM individually. Accordingly, differential effects of electroactive surface modulation on bacterial cells and ECM, at a gene regulatory level, can be deciphered. Better understanding of the formation and distribution of biofilms formed under environmental stress from electrical modulation of surface chemistries identifies ECM as important target to achieve biofilm eradication. The presented technique has potential to become fully automated, generating a high‐throughput analysis platform of surface materials and compounds designed to achieve biofilm eradication in medical and industrial context.

## 5 Experimental Section

### Surface Fabrication

ITO coated glass slides (75×25 mm) with surface resistivity of 70–100 Ω/sq (Merck, Germany) were cleaned by sonication for 20 min in acetone followed by 20 min in isopropanol using a Branson 6510 bath sonicator (Branson, USA). Slides were dried overnight at 150 °C. Oxygen Plasma cleaning of ITO coated slides was performed at mid RF for 20 min at 300–400 mTorr pressure using a PDC‐002‐HPCE Plasma Cleaner (Harrick Plasma, USA). PEDOT: PSS suspension for spin‐coating was prepared by mixing 10 ml Clevios PH1000 (Heraeus, Germany) with 200 εL glycerol and 25 εL sodium dodecylbenzenesulfonate while stirring with a magnetic rod. The dispersion was sonicated in a Branson 6510 bath sonicator for 20 min, then filtered through a 5 µm PVDF membrane filter (Merck, Germany). While stirring, 100 εL 3‐glycidyloxypropyl)trimethoxysilane (GOPS) was added. To cover the entire surface of cleaned ITO slides, ≈1500 εL of the dispersion was spin‐coated on each slide. Spin‐coating was done for initially 10 s (1 s acceleration + 9 s actual spinning) at 500 rpm, then for 46 s (1 s acceleration + 45 s actual spinning) at 1500 rpm using a WS‐650HZB‐23NPP/UD30 spin coater (Laurell technologies, USA). PEDOT:PSS coated slides were baked overnight at 150 °C. Before use, slides were dipped in sterile phosphate buffered saline (PBS) for 2.5 h for uniform wetting.

### Surface Characterization

For Scanning electron microscopy, dry surfaces were sputter coated with Pt using a Q150T ES Plus (Qourum Technologies, UK) to enhance the sample conductivity. Images were acquired using a Zeiss Gemini Ultra 55 scanning electron microscope equipped with on‐axis secondary electron detector (InLens, Sweden). Atomic force microscopy was performed using peak force tapping mode with Bruker Multimode 8 Atomic force microscopy and the analysis software Nanoscope Analyses version 1.5. Thickness measurement was performed using AFM by producing PEDOT:PSS/ITO slides using Kapton tape (Tesa 51 408) as mask. After removing the Kapton tape, the step size was measured. Wettability measurements were performed using a pocket goniometer (PGX, Fibro system AB, Sweden). The instrument automatically adds a water droplet of 2–5 µL to the surface and the contact angle was calculated by the software from the form of the droplet formed on the surface.

### Electrochemical Characterization of Electroactive Surfaces

Cyclic voltammetry was performed at 100 mV s^−1^ from −0.5 to 0.5 V or from −1.0 to 1.0 V with Gamry Reference 600 potentiostat (Gamry, USA) using a three‐electrode setup with a MW‐1033 Pt counter electrode (Basi Inc, USA) and a MF‐2056 Ag/AgCl reference electrode (Basi Inc, USA), filled with 3 m potassium chloride solution (Basi Inc, USA). Salt‐free Luria‐Bertani medium (LB medium) was used as electrolyte. Ten cycles were run and cycles 4–9 were averaged for each repeat. To calculate the charge stored within the electrochemical cell, the area inside the curve of the voltammograms was used. Chronoamperometry was performed using the same setup as above with a potential of 0.5 or 1.0 V and current was acquired for a total of 60 s in LB medium.

### Bacterial Strains and Culturing Conditions

The study used the wild‐type (wt) strain *Salmonella* serovar Enteritidis (*S*. Enteritidis) strain 3934 and isogenic mutants 3934 Δ*csgA::KmR* and 3934 *ΔcsgD::KmR*, all strains were transformed with the ampicillin‐resistant p2777 plasmid to enable stable GFP expression.^[^ ^21^ ^]^ Bacteria were revived from glycerol stocks by streaking them onto standard LB agar plates. Single colonies were streaked onto LB agar plates containing 50 µg mL^−1^ Ampicillin (Amp50). For each strain, one colony from the Amp50 plate was grown in LB medium without salt supplemented with 50 µg mL^−1^ Ampicillin over‐night at 37 °C under constant agitation. The overnight culture was diluted 1:100 in fresh LB medium without salt and grown for 2.5 h at 37 °C until mid‐exponential phase at OD 0.5. From the exponential phase culture, 30 ml was transferred to an autoclaved Coplin jar to set up the biofilm reactor.

### Biofilm Growth on Electroactive Surfaces in Biofilm Reactor

The biofilm reactor was set up in a biosafety cabinet to maintain sterile conditions. For running an experiment in whole‐cell mode, two sterile PEDOT:PSS/ITO slides were inserted into the Coplin jar containing the bacterial culture. The slides were positioned such that the coated side was facing the walls of the jar. When running the experiment in half‐cell mode, one PEDOT:PSS/ITO slide was inserted in the Coplin jar together with a Pt counter electrode (Basi Inc, USA). Alligator clips were connected to the inserted slides or electrodes. a custom‐made 3D printed autoclaved lid (3dVerkstan, Sweden) was used to close the jar, with care taken to safely secure the ends of the clips such that they were accessible for placing electrical leads. The biofilm reactor was immediately transferred to a 28 °C bacterial incubator and electrical leads were placed to connect the slides or electrodes to a Keithley 2602A source meter and bias potentials were applied. The biofilm reactor was incubated at 28 °C for 20 h to allow growth of *Salmonella* biofilm. During the full length of the incubation, surfaces and electrodes were constantly addressed with the bias potential and current was acquired using a GPIB‐USB interface (National Instruments, USA) and NI‐visa hardware control package (National Instruments, USA) and the Pyvisa package of the Python programming language. Data were smoothed by application of a Savitzky–Golay filter window length w  = 50 and a polynomial order of 2 using the SciPy package of the Python programming language.

### ECM Labeling

At the end of incubation, slides were withdrawn from the biofilm reactor with plastic tweezers and immediately mounted by spreading 200 µL phosphate buffered saline (PBS) containing 0.2 µL EbbaBiolight 680 (Ebba Biotech, Stockholm, Sweden) and placing a rectangular coverslip over the entire slide. Excess liquid was removed by leaning the slide toward a tissue. The coverslip was sealed with nail polish and carefully cleaned with tissue soaked in 70% ethanol prior to downstream analysis.

### Spectroscopic Mapping

Spectroscopic mapping was performed using an Infinite PRO1000 spectrophotometer (Tecan, Switzerland). Slides were placed faced‐down in a C4SH01 four‐slide holder (Thorlabs, USA) which had the same dimensions as standard microwell plates. The slide holder was placed on the plate reader stage. A protocol was set‐up that performed fluorescence readings in a 64 × 96 matrix with pixel dimensions of 1.3 mm x 1.3 mm. GFP fluorescence was detected using 488 nm excitation and 514 nm emission. EbbaBiolight 680 fluorescence was detected using 540 nm excitation and 650 nm emission. Fluorescence was plotted as colormap using Graph Pad Prism 10 (GraphPad Software, USA). Data for each repeat were averaged and presented as colormaps. The total amount of interface biofilm was calculated by averaging the rows of each colormap and calculating the AUC from the averaged column. Matlab and Python scripts assisting in colormap analysis are available at https://github.com/AIMES‐ARD/BiofilmAnalysis

### Microscopy

Confocal laser scanning microscopy of interface biofilms was performed with a Zeiss LSM900 confocal microscope at the Biomedicum Imaging Core (BIC) at Karolinska Institutet, Solna. GFP was excited at 488 nm and an emission filter with 410–546 nm bandwidth was chosen. EbbaBiolight 680 fluorescence was excited with 561 nm and an emission filter with 595–700 nm bandwidth was chosen. Interface biofilms were imaged at 10X magnification and a total of ten images with 5 rows and 2 columns were acquired. Each image contained 10–20 focal planes through the biofilm in depth (z) with 1.98 µm step size. Image processing was performed using ImageJ 1.54f (NIH, USA) as well as aicsimageio, numpy, scipy and pandas packages of the Python programming language and Matlab computing platform (Mathworks, USA). Matlab and Python scripts for high‐resolution, large‐area image analysis are available at https://github.com/AIMES‐ARD/BiofilmAnalysis Graphs were plotted using Graph Pad Prism 10 (GraphPad Software, USA).

## Conflict of Interest

S. Löffler (SL) and A. Richter‐Dahlfors (A.R.D.) are co‐inventors of patents relevant to this work. Intellectual properties are owned by Richter Life Science Development AB, founded by A.R.D.. S.L. and A.R.D. have engagement in Ebba Biotech AB, which commercializes optotracers for uses as described in this article.

## Supporting information

Supporting Information

## Data Availability

The data that support the findings of this study are available from the corresponding author upon reasonable request.

## References

[1] H.‐C. Flemming , T. R. Neu , D. J. Wozniak , J. Bacteriol. 2007, 189, 7945. 17675377 10.1128/JB.00858-07 PMC2168682

[2] H.‐C. Flemming , S. Wuertz , Nat. Rev. Microbiol. 2019, 17, 247. 30760902 10.1038/s41579-019-0158-9

[3] A. Penesyan , I. T. Paulsen , S. Kjelleberg , M. R. Gillings , NPJ Biofilms Microbiomes 2021, 7, 80. 34759294 10.1038/s41522-021-00251-2 PMC8581019

[4] D. H. Limoli , C. J. Jones , D. J. Wozniak , Microbiol. Spectr. 2015, 3, 10.1128/microbiolspec.mb-0011-2014. PMC4657554 26185074

[5] A. Dragos , Á. T. Kovács , Trends Microbiol. 2017, 25, 257. 28089324 10.1016/j.tim.2016.12.010

[6] A. Gupta , R. Gupta , R. L. Singh , Principles and Application of Environmental Biotechnology for a Sustainable Future, Springer, Singapore 2017, pp. 43.

[7] L. Hall‐Stoodley , J. W. Costerton , P. Stoodley , Nat. Rev. Microbiol. 2004, 2, 95. 15040259 10.1038/nrmicro821

[8] T. Bjarnsholt , M. Whiteley , K. P. Rumbaugh , P. S. Stewart , P. Ø. Jensen , N. Frimodt‐Møller , Lancet Infect. Dis. 2022, 22, e88. 34506737 10.1016/S1473-3099(21)00122-5 PMC9190128

[9] T. Bjarnsholt , APMIS 2013, 121, 1. 23635385 10.1111/apm.12099

[10] H.‐C. Flemming , Water Res. 2020, 173, 115576. 32044598 10.1016/j.watres.2020.115576

[11] A. Bridier , P. Sanchez‐Vizuete , M. Guilbaud , J.‐C. Piard , M. Naïtali , R. Briandet , Food Microbiol. 2015, 45, 167. 25500382 10.1016/j.fm.2014.04.015

[12] A.‐M. Holban , C. Farcasiu , O.‐C. Andrei , A. M. Grumezescu , A.‐T. Farcasiu , Materials 2021, 14, 6994. 34832390 10.3390/ma14226994 PMC8625127

[13] S. Gomez‐Carretero , B. Libberton , K. Svennersten , K. Persson , E. Jager , M. Berggren , M. Rhen , A. Richter‐Dahlfors , NPJ Biofilms Microbiomes 2017, 3, 10.1038/s41522-017-0027-0. PMC6081481 30109118

[14] H. Yamato , M. Ohwa , W. Wernet , J. Electroanal. Chem. 1995, 397, 163.

[15] E.‐G. Kim , J.‐L. Brédas , J. Am. Chem. Soc. 2008, 130, 16880. 19053439 10.1021/ja806389b

[16] K. Butina , S. Löffler , M. Rhen , A. Richter‐Dahlfors , Sens. Actuators B: Chem. 2019, 297, 126703.

[17] P. J. Wood , Carbohydr. Res. 1980, 85, 271.

[18] U. Römling , Cell. Mol. Life Sci. 2005, 62, 1234. 15818467 10.1007/s00018-005-4557-x PMC11139082

[19] M. Strathmann , J. Wingender , H.‐C. Flemming , J. Microbiol. Methods 2002, 50, 237. 12031574 10.1016/s0167-7012(02)00032-5

[20] P. Larsen , J. L. Nielsen , M. S. Dueholm , R. Wetzel , D. Otzen , P. H. Nielsen , Environ. Microbiol. 2007, 9, 3077. 17991035 10.1111/j.1462-2920.2007.01418.x

[21] F. X. Choong , M. Bäck , S. Fahlén , L. B. Johansson , K. Melican , M. Rhen , K. P. R. Nilsson , A. Richter‐Dahlfors , NPJ Biofilms Microbiomes 2016, 2, 16024. 28721253 10.1038/npjbiofilms.2016.24 PMC5515270

[22] F. X. Choong , S. Huzell , M. Rosenberg , J. A. Eckert , M. Nagaraj , T. Zhang , K. Melican , D. E. Otzen , A. Richter‐Dahlfors , Biofilm 2021, 3, 100060. 34841245 10.1016/j.bioflm.2021.100060 PMC8605384

[23] H. Antypas , T. Zhang , F. X. Choong , K. Melican , A. Richter‐Dahlfors , FEMS Microbes 2023, 4, xtad007. 37333433 10.1093/femsmc/xtad007 PMC10117878

[24] J. A. Eckert , M. Rosenberg , M. Rhen , F. X. Choong , A. Richter‐Dahlfors , Biofilm 2022, 4, 100083. 36117547 10.1016/j.bioflm.2022.100083 PMC9474290

[25] E. Kärkkäinen , S. G. Jakobsson , U. Edlund , A. Richter‐Dahlfors , F. X. Choong , Front. Cell. Infect. Microbiol. 2022, 12, 981454. 36118028 10.3389/fcimb.2022.981454 PMC9478205

[26] B. Coppens , T. E. R. Belpaire , J. Pešek , H. P. Steenackers , H. Ramon , B. Smeets , iScience 2023, 26, 106861. 37260744 10.1016/j.isci.2023.106861 PMC10227381

[27] A. Rodríguez‐Rojas , D. Y. Baeder , P. Johnston , R. R. Regoes , J. Rolff , PLoS Pathog. 2021, 17, e1009443. 33788905 10.1371/journal.ppat.1009443 PMC8041211

[28] K. Lorenz , L. Preem , K. Sagor , M. Putrins , T. Tenson , K. Kogermann , Mol. Pharmaceutics 2023, 20, 1230. 10.1021/acs.molpharmaceut.2c00902 PMC9907351 36669095

[29] A. Sass , I. Vandenbussche , B. Bellich , P. Cescutti , T. Coenye , J. Bacteriol. 2022, 204, e00017. 35416687 10.1128/jb.00017-22 PMC9112949

[30] L. H. P. Pham , M. Colon‐Ascanio , J. Ou , K. Ly , P. Hu , J. S. Choy , X. Luo , Lab Chip 2022, 22, 4349. 36239125 10.1039/d2lc00728b PMC9756269

[31] P. Merkl , M.‐S. Aschtgen , B. Henriques‐Normark , G. A. Sotiriou , Biosens. Bioelectron. 2021, 171, 112732. 33120233 10.1016/j.bios.2020.112732 PMC7116521

[32] S. K. Hood , E. A. Zottola , Int. J. Food Microbiol. 1997, 37, 145. 9310849 10.1016/s0168-1605(97)00071-8

[33] U. Römling , W. D. Sierralta , K. Eriksson , S. Normark , Mol. Microbiol. 1998, 28, 249. 9622351 10.1046/j.1365-2958.1998.00791.x

[34] A. M. Prouty , J. S. Gunn , Infect. Immun. 2003, 71, 7154. 14638807 10.1128/IAI.71.12.7154-7158.2003 PMC308894

[35] Y. B. Ngwai , Y. Adachi , Y. Ogawa , H. Hara , J. Microbiol., Immunol. Infect. 2006, 39, 189. 16926973

[36] S. Gomez‐Carretero , R. Nybom , A. Richter‐Dahlfors , Adv. Healthcare Mater. 2017, 6, 1700435. 10.1002/adhm.201700435 28805046

[37] I. W. Sutherland , Trends Microbiol. 2001, 9, 222. 11336839 10.1016/s0966-842x(01)02012-1

[38] H.‐C. Flemming , J. Wingender , Nat. Rev. Microbiol. 2010, 8, 623. 20676145 10.1038/nrmicro2415

[39] C. Solano , B. García , J. Valle , C. Berasain , J.‐M. Ghigo , C. Gamazo , I. Lasa , Mol. Microbiol. 2002, 43, 793. 11929533 10.1046/j.1365-2958.2002.02802.x

[40] J. W. Costerton , Z. Lewandowski , D. E. Caldwell , D. R. Korber , H. M. Lappin‐Scott , Annu. Rev. Microbiol. 1995, 49, 711. 8561477 10.1146/annurev.mi.49.100195.003431

[41] U. Römling , D. Pesen , S. Yaron , in Salmonella: Molecular Biology and Pathogenesis (Eds: M. Rhen , D. Maskell , P. Mastroeni , J. Therfall ), Horizon Bioscience, Norwich, UK 2007, Ch. 7

[42] J. Jantsch , C. Cheminay , D. Chakravortty , T. Lindig , J. Hein , M. Hensel , Cell. Microbiol. 2003, 5, 933. 14641178 10.1046/j.1462-5822.2003.00334.x

[43] A. Håkansson , S. Han , S. Wang , J. Lu , S. Braun , M. Fahlman , M. Berggren , X. Crispin , S. Fabiano , J. Polym. Sci. B: Polym. Phys. 2017, 55, 814.

[44] A. V. Volkov , K. Wijeratne , E. Mitraka , U. Ail , D. Zhao , K. Tybrandt , J. W. Andreasen , M. Berggren , X. Crispin , I. V. Zozoulenko , Adv. Funct. Mater. 2017, 27, 1700329.

[45] J. Lipus , K. Krukiewicz , Measurement 2022, 191, 110822.

[46] K. Butina , S. Löffler , M. Rhen , A. Richter‐Dahlfors , Sens. Actuators B: Chem. 2019, 297, 126703.

[47] W. Yang , A. Broski , J. Wu , Q. H. Fan , W. Li , IEEE Trans. Nanotechnol. 2018, 17, 701. 30745860 10.1109/TNANO.2017.2785627 PMC6368347

[48] A. Broski , Y. Guo , W. A. Khan , W. Li , in IEEE 12th Int. Conf. on Nano/Micro Engineered and Molecular Systems, NEMS 2017, Los Angeles, CA, USA 2017.

[49] Y. A. Gorby , S. Yanina , J. S. Mclean , K. M. Rosso , D. Moyles , A. Dohnalkova , T. J. Beveridge , I. S. Chang , B. H. Kim , K. S. Kim , D. E. Culley , S. B. Reed , M. F. Romine , D. A. Saffarini , E. A. Hill , L. Shi , D. A. Elias , D. W. Kennedy , G. Pinchuk , K. Watanabe , S. Ishii , B. Logan , K. H. Nealson , J. K. Fredrickson , Proc. Natl. Acad. Sci. USA 2006, 103, 11358. 16849424 10.1073/pnas.0604517103 PMC1544091

[50] F. J. R. Meysman , Trends Microbiol. 2018, 26, 411. 29174100 10.1016/j.tim.2017.10.011

[51] C. I. Torres , A. Kato Marcus , B. E. Rittmann , Biotechnol. Bioeng. 2008, 100, 872. 18551519 10.1002/bit.21821

[52] A. Turolla , M. Di Mauro , L. Mezzera , M. Antonelli , M. Carminati , Sens. Actuators B: Chem. 2019, 281, 288.

[53] J. Paredes , M. Alonso‐Arce , C. Schmidt , D. Valderas , B. Sedano , J. Legarda , F. Arizti , E. Gómez , A. Aguinaga , J. L. Del Pozo , S. Arana , Biomed. Microdevices 2014, 16, 365. 24515846 10.1007/s10544-014-9839-3

[54] A. Shafaat , J. F. Gonzalez‐Martinez , W. O. Silva , A. Lesch , B. Nagar , Z. da Silva , J. Neilands , J. Sotres , S. Björklund , H. Girault , T. Ruzgas , Angew. Chem., Int. Ed. 2023, 62, e202308181. 10.1002/anie.202308181 37490019

[55] K. Butina , F. Filipovic , A. Richter‐Dahlfors , O. Parlak , Adv. Mater. Interfaces 2021, 8, 2100961.

[56] W. Zhang , E. S. Mclamore , N. T. Garland , J. V. C. Leon , M. K. Banks , J. Microbiol. Methods 2013, 94, 367. 23916866 10.1016/j.mimet.2013.07.022

[57] Z. Sanchez , A. Tani , K. Kimbara , Appl. Environ. Microbiol. 2013, 79, 1396. 23220960 10.1128/AEM.02911-12 PMC3568627

[58] Y.‐J. Tsai , Y.‐C. Lin , W.‐B. Wu , P.‐H. Chiu , B. J. Lin , S.‐P. Hao , Otolaryngol. – Head Neck Surg. 2013, 148, 633. 23348872 10.1177/0194599812474971

[59] M. A. Olszewska , A. Gedas , M. Simões , Molecules 2020, 25, 2641. 32517201 10.3390/molecules25112641 PMC7321256

[60] S. A. Waller , A. I. Packman , M. Hausner , J. Microbiol. Methods 2018, 144, 8. 29111400 10.1016/j.mimet.2017.10.013

[61] S. Rogers , K. Honma , T. S. Mang , Photodiagnosis Photodyn. Ther. 2018, 23, 18. 29753881 10.1016/j.pdpdt.2018.04.015

[62] L. Johnson , S. R. Horsman , L. Charron‐Mazenod , A. L. Turnbull , H. Mulcahy , M. G. Surette , S. Lewenza , BMC Microbiol. 2013, 13, 115. 23705831 10.1186/1471-2180-13-115 PMC3724500

[63] K. A. Gränicher , L. Karygianni , T. Attin , T. Thurnheer , Front. Microbiol. 2021, 12, 741863. 34650542 10.3389/fmicb.2021.741863 PMC8506149

[64] S. Ravaioli , D. Campoccia , P. Speziale , G. Pietrocola , B. Zatorska , A. Maso , E. Presterl , L. Montanaro , C. R. Arciola , Biofouling 2020, 36, 86. 31985269 10.1080/08927014.2020.1716217

[65] H. Ogasawara , K. Yamamoto , A. Ishihama , J. Bacteriol. 2011, 193, 2587. 21421764 10.1128/JB.01468-10 PMC3133154

[66] A. S. Sokaribo , E. G. Hansen , M. Mccarthy , T. S. Desin , L. L. Waldner , K. D. Mackenzie , G. Mutwiri , N. J. Herman , D. J. Herman , Y. Wang , A. P. White , Microorganisms 2020, 8, 964. 32604994 10.3390/microorganisms8070964 PMC7409106

[67] U. Gerstel , U. Römling , Res. Microbiol. 2003, 154, 659. 14643403 10.1016/j.resmic.2003.08.005

