# Supplementary material for: High‐Resolution Large‐Area Image Analysis Deciphers the Distribution of Salmonella Cells and ECM Components in Biofilms Formed on Charged PEDOT:PSS Surfaces
Source: Adv Sci (Weinh). 2024 Jan 15;11(27):2307322. doi: 10.1002/advs.202307322 (PMC11251553; doi:10.1002/advs.202307322)
Supplement: Supplementary file 1 — Supporting Information [file ADVS-11-2307322-s001.pdf]

ADVANCED SCIENCE
Open Access

Supporting Information

for *Adv. Sci.*, DOI 10.1002/advs.202307322

High-Resolution Large-Area Image Analysis Deciphers the Distribution of *Salmonella* Cells and ECM Components in Biofilms Formed on Charged PEDOT:PSS Surfaces

Sanhita Ray, Susanne Löffler and Agneta Richter-Dahlfors**

Supporting Information

High-resolution large-area image analysis deciphers the distribution of *Salmonella* cells and ECM components in biofilms formed on charged PEDOT:PSS surfaces

Sanhita Ray, Susanne Löffler, Agneta Richter-Dahlfors**

* Corresponding Authors

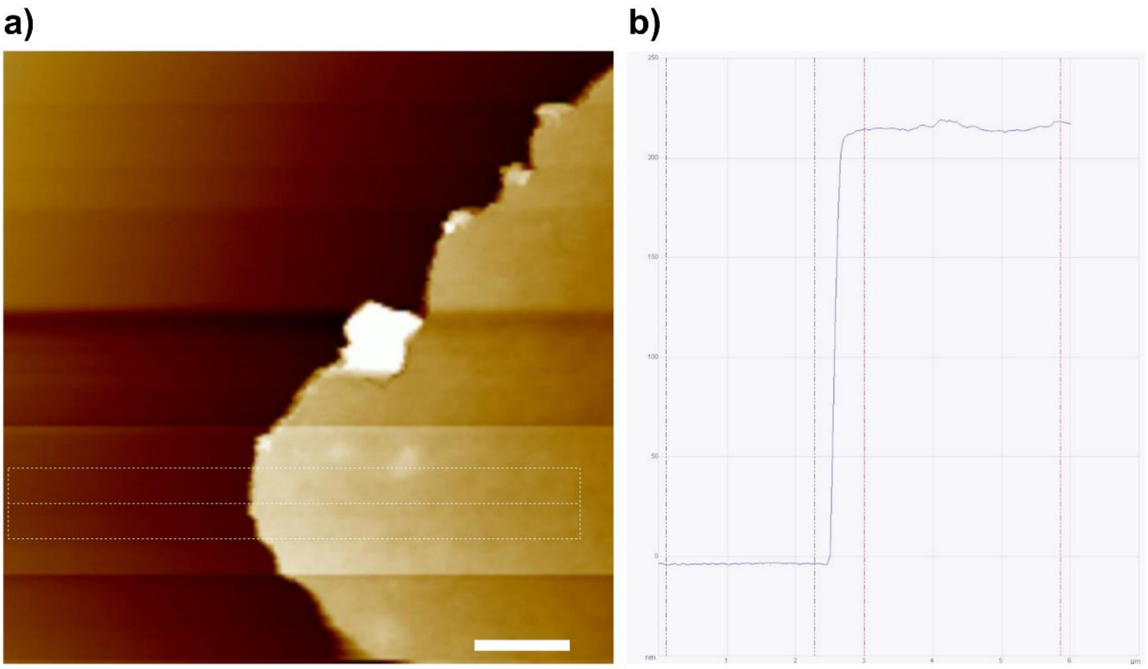

Figure S1. Thickness of PEDOT:PSS coated on ITO slide. (**a**) AFM scan of the interface between an uncoated area on the ITO slide and an area coated with PEDOT:PSS. Scale bar = 1 µm. (**b**) The step height (vertical axis) from uncoated to coated area along the horizontal axis.

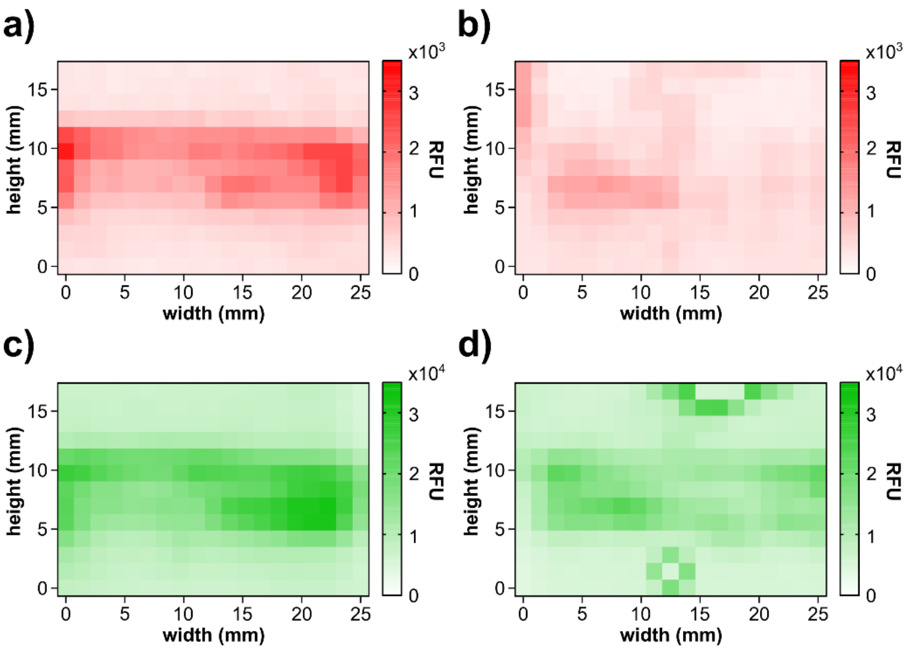

Figure S2. Spectroscopic mapping of *Salmonella* wt-GFP biofilm formed on electroactive surfaces addressed in the whole-cell setup at 1.0 V bias potential. Averaged results for spectroscopic mapping shown as colormaps of *Salmonella* wt-GFP biofilm, formed on (**a, c**) the oxidized and (**b, d**) the reduced PEDOT:PSS/ITO slide in the whole-cell setup at 1.0 V bias potential. The colormaps show the average fluorescence intensity (RFU) of (**a, b**) EbbaBiolight 680 (red) representing the ECM, and (**c, d**) GFP (green) representing bacterial cells. Results of the colormap-quantification are shown in Figure 4c and Figure 4f.

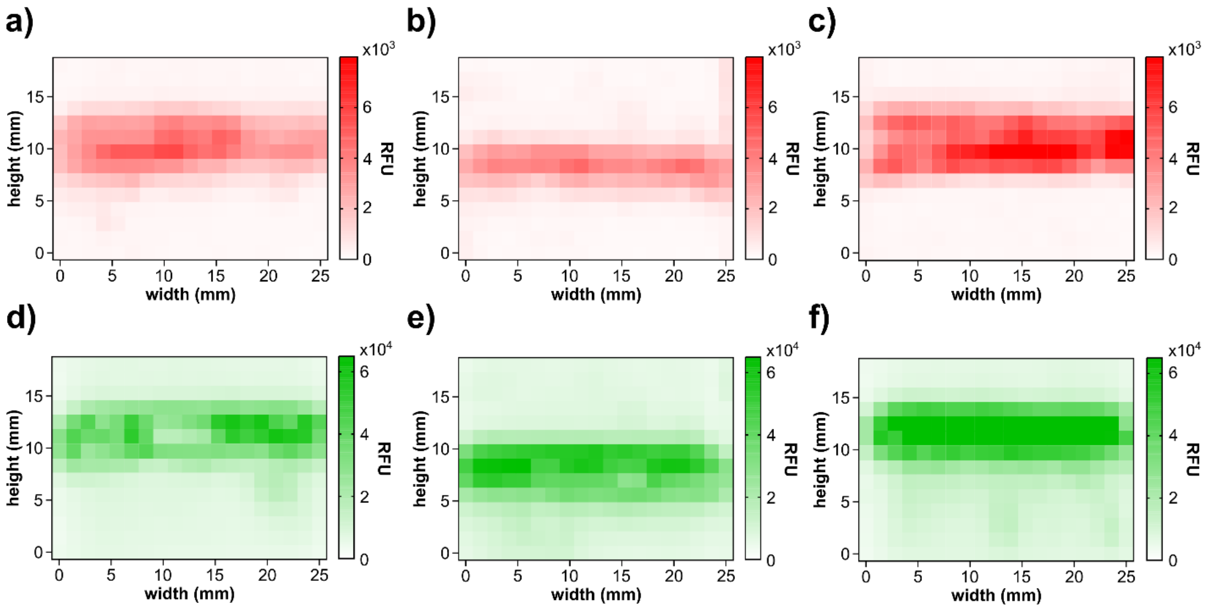

Figure S3. Spectroscopic mapping of *Salmonella* wt-GFP biofilm in the half-cell setup. Averaged results for spectroscopic mapping shown as colormaps of *Salmonella* wt-GFP biofilm formed on PEDOT:PSS/ITO slides in the half-cell setup. Colormaps are shown for (**a, d**) oxidized, (**b, e**) unbiased and (**c, f**) reduced surfaces obtained by applying ±0.5 V bias potential with respect to a Pt counter electrode, or unbiased (open circuit) with respect to a Pt counter electrode. The colormaps show the average fluorescence intensity (RFU) of (**a-c**) EbbaBiolight 680 (red) representing the ECM, and (**d-f**) GFP (green) representing bacterial cells. Results of the colormap-quantifications are shown in Figure 3h.31

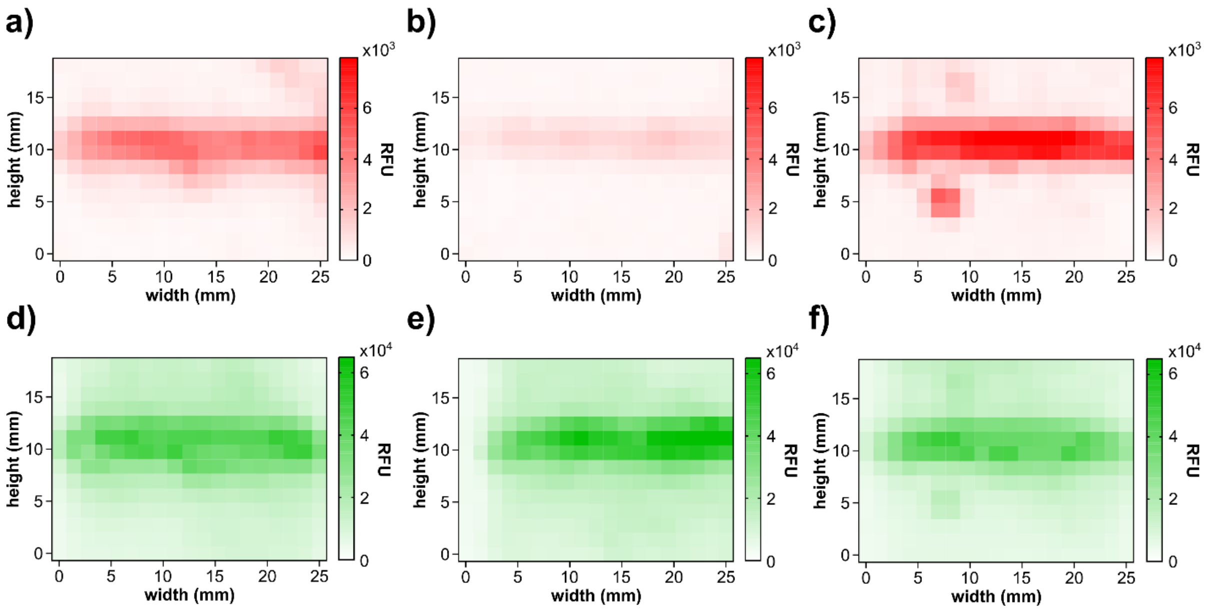

Figure S4. Spectroscopic mapping of *Salmonella ΔcsgA*-GFP biofilm in the half-cell setup. Averaged results for spectroscopic mapping shown as colormaps of *Salmonella ΔcsgA*-GFP biofilm formed on PEDOT:PSS/ITO slides in the half-cell setup. Colormaps are shown for (**a, d**) oxidized, (**b, e**) unbiased and (**c, f**) reduced surfaces obtained by applying ±0.5 V bias potential with respect to a Pt counter electrode, or unbiased (open circuit) with respect to a Pt counter electrode. The colormaps show the average fluorescence intensity (RFU) of (**a-c**) EbbaBiolight 680 (red) representing the ECM, and (**d-f**) GFP (green) representing bacterial cells. Results of the colormap-quantifications are shown in Figure 3i.

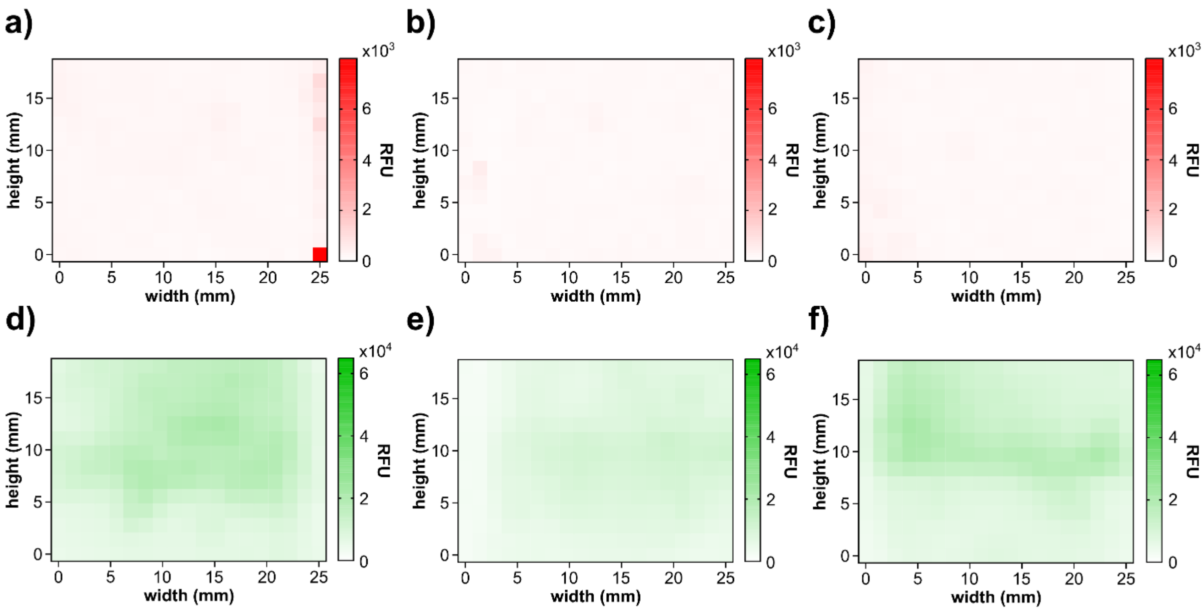

Figure S5. Spectroscopic mapping of *Salmonella ΔcsgD*-GFP biofilm in the half-cell setup. Averaged results for spectroscopic mapping shown as colormaps of *Salmonella ΔcsgD*-GFP biofilm formed on PEDOT:PSS/ITO slides in the half-cell setup. Colormaps are shown for (**a, d**) oxidized, (**b, e**) unbiased and (**c, f**) reduced surfaces obtained by applying ±0.5 V bias potential with respect to a Pt counter electrode, or unbiased (open circuit) with respect to a Pt counter electrode. The colormaps show the average fluorescence intensity (RFU) of (**a-c**) EbbaBiolight 680 (red) representing the ECM, and (**d-f**) GFP (green) representing bacterial cells. Results of the colormap-quantifications are shown in Figure 3j.
